# Supplementary material for: Causal interplay between lactose intolerance and gut microbiota: a combined bidirectional Mendelian randomization and in vivo validation study
Source: Front Nutr. 2026 Jun 1;13:1803337. doi: 10.3389/fnut.2026.1803337 (PMC13265576; doi:10.3389/fnut.2026.1803337)
Supplement: Supplementary file 1 [file Data_Sheet_1.zip › supplementary materials/Forward/funnel chart/ebi-a-GCST90027736.finngen_R12_E4_LACTONAS.pdf]

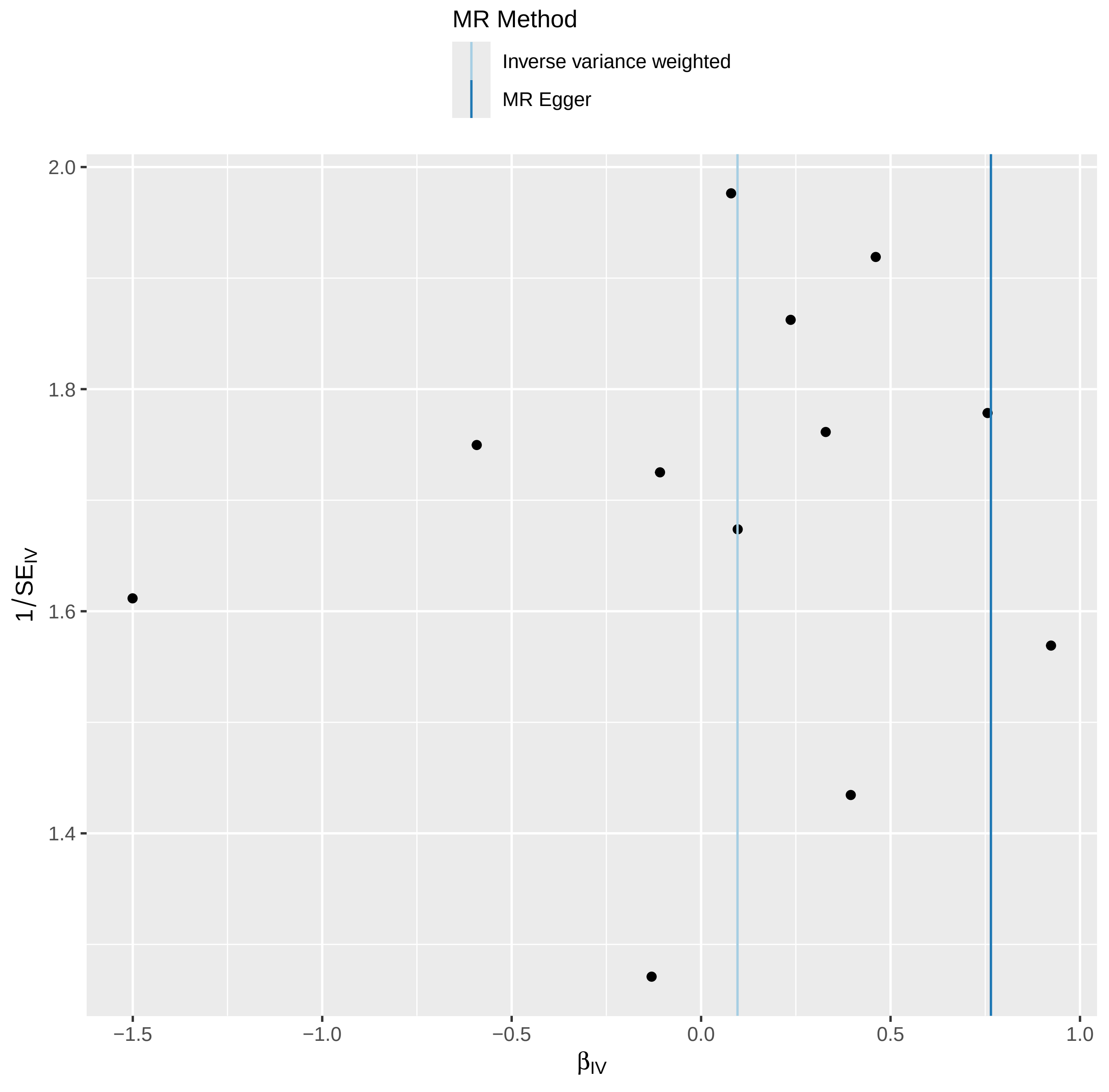

MR Method
Inverse variance weighted
MR Egger
2.0
1.8
1.6
1.4
$1/SE_{IV}$
−1.5
−1.0
−0.5
0.0
0.5
1.0
β_{IV}
